# Sound Quality Factors Inducing the Autonomous Sensory Meridian Response

**DOI:** 10.3390/audiolres12050056

**Published:** 2022-10-13

**Authors:** Ryota Shimokura

**Affiliations:** Graduate School of Engineering Science, Osaka University, Room D436, 1-3 Machikaneyama, Toyonaka 560-8531, Japan; rshimo@sys.es.osaka-u.ac.jp; Tel./Fax: +81-6-6850-6376

**Keywords:** autonomous sensory meridian response, loudness, roughness, interaural cross-correlation coefficient

## Abstract

The acoustical characteristics of auditory triggers often recommended to generate the autonomous sensory meridian response (ASMR) on Internet platforms were investigated by parameterizing their sound qualities following Zwicker’s procedure and calculating autocorrelation (ACF)/interaural cross-correlation (IACF) functions. For 20 triggers (10 human- and 10 nature-generated sounds), scores (on a five-point Likert scale) of the ASMR, perceived loudness, perceived pitch, comfort, and perceived closeness to the sound image were obtained for 26 participants by questionnaire. The results show that the human-generated sounds were more likely to trigger stronger ASMR than nature-generated sounds, and the primary psychological aspect relating to the ASMR was the perceived closeness, with the triggers perceived more closely to a listener having higher ASMR scores. The perceived closeness was evaluated by the loudness and roughness (among Zwicker’s parameter) for the nature-generated sounds and the interaural cross-correlation coefficient (IACC) (among ACF/IACF parameters) for the human-generated sounds. The nature-generated sounds with higher loudness and roughness and the human-generated sounds with lower IACC were likely to evoke the ASMR sensation.

## 1. Introduction

The autonomous sensory meridian response (ASMR) is an atypical sensory phenomenon in which individuals experience a tingling, static sensation across the scalp and back of the neck in response to specific triggering audio and visual stimuli or to light touch [1]. This sensation is widely reported to promote relaxation, wellbeing, and sleep, and there are many ASMR-related channels on YouTube. Some researchers have examined the relationship between the ASMR and misophonia [2,3,4]. Misophonia is an auditory disorder of decreased tolerance to specific sounds or their associated stimuli such as oral sounds (e.g., loud breathing, chewing, swallowing), clicking sounds (e.g., keyboard tapping, finger tapping, windshield wipers), and sounds associated with movement (e.g., fidgeting) [5,6,7,8]. The ASMR triggers produce positive emotions associated with an increase of wellbeing, while the misophonia triggers produce negative emotions associated with fight-or-flight responses. Although the displayed emotions are opposite, both are caused commonly by hypersensitivities to sound triggers, and it is possible that the acoustical characteristics of the ASMR triggers may explain the occurrence mechanism of the misophonia. Actually, a previous study reported that people who experienced the ASMR were more likely to have a risk of misophonia [2].

Several common audio and visual stimuli (triggers) that induce the ASMR are known, and an online ASMR experience questionnaire completed by 475 individuals identified the trigger types as whispering (75%), personal attention (69%), crisp sounds (64%), and slow movements (53% participants reporting the ASMR experience) [1]. Following this questionnaire, many studies on the ASMR have empirically selected such highly possible triggers [9,10,11,12,13]. However, it is not clear which physical characteristics of these triggers induce the ASMR.

In the case of audio signals, numerical models have been proposed to define the sound quality. Perceptual characteristics of the hearing of sound are the loudness, pitch, and timbre, and the sound quality is expressed generally by numerical algorithms based on varying sound pressure. As an example, Zwicker’s parameters (loudness, sharpness, roughness, and fluctuation strength) have been used to evaluate the sound quality of environmental noise [14]. The loudness is the psychological sound intensity, and it is calculated by transforming the frequency onto the Bark scale, considering the effects of frequency and temporal masking, and counting the area of the loudness pattern [15]. The loudness of a pure tone with a frequency of 1 kHz and sound pressure level of 40 dB is defined as being 1 sone. The sharpness is a measure of the sound acuity and high-frequency component, and is obtained by adding a weight function to its specific loudness [16]. The sharpness of a noise at 60 dB in a critical band at 1 kHz is defined as being 1 acum. The roughness is a fundamental hearing sensation caused by sound with rapid amplitude modulation (15–300 Hz) and is quantified on the basis of the modulation frequency and depth of the time-varying loudness [16]. The roughness of a 1 kHz tone at 60 dB with a 100% amplitude modulation (modulation depth of 1) at 70 Hz is defined as being 1 asper. The fluctuation strength is similar in principle to roughness except that it quantifies the subjective perception of the slower (up to 20 Hz) amplitude modulation of a sound, and it is calculated from the modulation frequency and depth of the time-varying loudness [16]. The fluctuation strength produced by a 1 kHz tone at 60 dB with a 100% amplitude modulated at 4 Hz is defined as being 1 vacil.

The other procedure for evaluating sound quality is using the autocorrelation and interaural cross-correlation functions (ACF and IACF) frequently used for music and acoustics in concert halls [17]. Our auditory perceptions are deeply related to the timing of nerve firings caused by binaurally detected sounds, and the ACF and IACF are modeled in the processors of the auditory nerve [18,19]. Three parameters can be calculated from ACF analyses of monoaurally recorded sound: (1) the delay time of the maximum peak (τ_1_), (2) the amplitude of the first maximum peak (ϕ_1_) and (3) the width of the peak at the original time [W_Φ(0)_] (see Section 2.2 for details). The fundamental frequency (1/τ_1_ Hz) and the pitch strength of the sound are τ_1_ and ϕ_1_, respectively. The spectral centroid of the original signal is W_Φ(0)_, with longer and shorter values, respectively, corresponding to lower and higher centroid values of spectral energy signals. These ACF parameters explain not only the musical motif suitable for a specific concert hall [17] but also annoyance induced by noise [20,21] and speech intelligibility [22,23]. From the IACF analyses of binaurally recorded sound, the interaural cross-correlation coefficient (IACC) can be calculated (see Section 2.1 for details). The IACC is the maximum peak amplitude of the IACF whose delay time is within ±1 ms. The IACC is related to the subjective sound diffuseness, which means that a higher IACC corresponds to the listener perceiving a well-defined direction of the incoming sound, whereas a lower IACC corresponds to a well-diffused sound. Such ACF and IACF parameters have also been used for the evaluation of several types of noise [24,25,26,27].

The present study identified physical factors that induce the auditory-based ASMR sensation using the four Zwicker parameters and four ACF/IACF parameters. We prepared a total of 20 sound motifs likely to induce the ASMR and calculated the eight sound quality parameters. To confirm the occurrence of the ASMR, previous studies have adopted physiological (e.g., functional magnetic resonance imaging or heat rate) [11,28,29] and psychological (e.g., questionaries) [1,9,10,12,13] procedures. The present study adopted the psychological approach, with participants quantifying the degree of the perceived ASMR on a five-point Likert scale. In addition to the ASMR, the participants scored four subjective sensations (subjective loudness, pitch, comfort, and closeness) at the same time. We examined the correlation of the ASMR scores with the four subjective sensations and eight sound quality parameters.

## 2. Method

### 2.1. ASMR Triggers and Sound Quality Parameters

The 10 auditory ASMR triggers (human-generated sounds) used in the study, and 10 healing sounds (nature-generated sounds) recorded binaurally were added for the comparison (Table 1). The human- and nature-generated sounds were obtained from several websites and music distribution sites, respectively. The human-generated sounds were recorded by a dummy head microphone or a binaurally wearing microphone. Although the nature-generated sounds do not have information on the recording devices, the participants of this study could perceive the sound images close to them with binaural hearing. For the sake of expediency, both sounds are called as trigger. The human- and nature-generated sounds, respectively, represent sounds generated by human behaviors (e.g., the cutting of vegetables and typing at a keyboard) and natural phenomena (e.g., waves and rain). The time length of each trigger was 50 s, and the sound energy was set at the same equivalent continuous A-weighted sound pressure level (*L*_Aeq_) of 45 dBA.

Table 1 lists the sound quality parameters. The Zwicker parameters were calculated using a Matlab command embedded in Auditory Toolbox [30]. The calculation algorithms were based on work in the literature [14,15,16]. The calculations of roughness and fluctuation strength had running steps of 0.5 ms and 2 ms, respectively, along the time length of 50 s, and Table 1 lists average values of the time-varying parameters.

The ACF parameters were calculated from the normalized ACF:

(1) $$\phi_{ll}\left(\tau \right)=\phi_{ll}\left(\tau ;s,T\right)=\frac{\Phi_{ll}\left(\tau ;s,T\right)}{\Phi_{ll}\left(0;s,T\right)},$$

where

(2) $$\Phi_{ll}\left(\tau ;s,T\right)=\frac{1}{2T}\int_{s-T}^{s+T}{p_{l}}^{\prime }\left(t\right){p_{l}}^{\prime }\left(t+\tau \right)dt.$$

**Figure 1:**
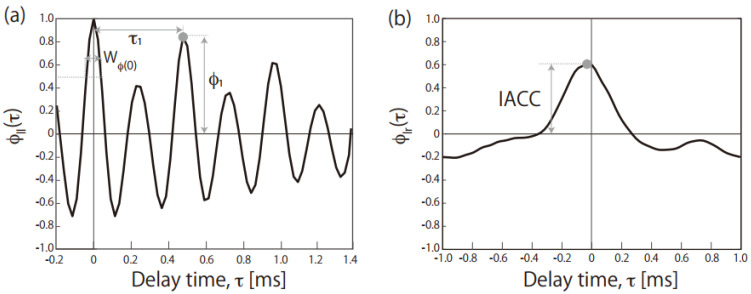
(**a**) Normalized ACF of *Cicada* as a nature-generated sound and (**b**) normalized IACF of *Cutting* as a human-generated sound. The definitions of τ_1_, ϕ_1_, W_Φ(0)_ and the IACC are included.

Here, *τ* is the delay time [s], *s* is the running step [s], 2*T* is the integration interval [s] and *p*_l_′(*t*) is the sound in the left channel at time *t* after passing through an A-weighted network. The ACF parameters were the (1) delay time of the maximum peak (τ_1_), (2) amplitude of the first maximum peak (ϕ_1_) and (3) width of the peak at τ = 0 (W_Φ(0)_), calculated by doubling the delay time at which the normalized ACF becomes 0.5 times that at the origin of the delay (Figure 1a). Additionally, τ_1_ and ϕ_1_ are related to the pitch (high or low) and pitch strength (clear or ambiguous) perceived in the periodical part of the sound. The spectral centroid is equivalent to W_Φ__(0)_, and a sound with greater W_Φ__(0)_ is thus perceived as having a lower pitch in the noisy part.

The IACC was calculated from the normalized IACF:

(3) $$\phi_{lr}\left(\tau \right)=\phi_{lr}\left(\tau \right)=\frac{\Phi_{lr}\left(\tau ;s,T\right)}{\sqrt{\Phi_{ll}\left(0;s,T\right)\Phi_{rr}\left(0;s,T\right)}},\;$$

where

(4) $$\Phi_{lr}=\frac{1}{2T}\int_{s-T}^{s+T}{p_{l}}^{\prime }\left(t\right){p_{r}}^{\prime }\left(t+\tau \right)dt.\;$$

Here, Φ_rr_ is the ACF for the right channel and *p*_r_′(*t*) is the A-weighted sound in the right channel. The IACC is the maximum peak amplitude of the IACF whose delay time is within ±1 ms (Figure 1b). The IACC is related to the subjective sound diffuseness mentioned in the Introduction. The integration interval (2*T*) and running step (*s*) were, respectively, 1 and 0.5 s for the both ACF and IACF calculations, and Table 1 lists average values of the time-varying parameters.

### 2.2. Participants

We recruited 26 participants (20 men and 6 women; age: 21.7 ± 0.4 years) who had normal hearing. All participants self-reported that they knew of the ASMR through watching Japanese YouTube channels. The institutional ethics committee approved the experimental protocol (approval code: R3-19).

### 2.3. Tasks and Procedures

After listening to the ASMR trigger (50 s) through headphones (HD598, Sennheiser, Wedemark, Germany) binaurally, the participants were instructed to provide scores on a five-point Likert scale in the subsequent 10 s. The *L*_Aeq_ at the ear positions was adjusted to 45 dBA. After mounting the headphones on a head and torso simulator (type 4128; Brüel & Kjær, Naerum, Denmark), the output level was adjusted to the 45 dBA in the average of the left and right channels. The participants were asked to give scores (−2, −1, 0, 1 or 2) for the degree of perceived loudness (from −2: not so loud to 2: very loud), perceived pitch (from −2: very low to 2: very high), comfort (from −2: not so comfortable to 2: very comfortable), perceived closeness to the sound image (from −2: very far to 2: very close) and ASMR (from −2: not feeling an ASMR to 2: feeling a strong ASMR) on the question sheet. The order of presentation of the AMSR triggers was randomized. The experiment was conducted in an anechoic chamber (*L*_Aeq_ of the background noise below 30 dB) at Osaka University, Japan. The Matlab was used to calculate the statistical values in the following section.

## 3. Results

**Figure 2:**
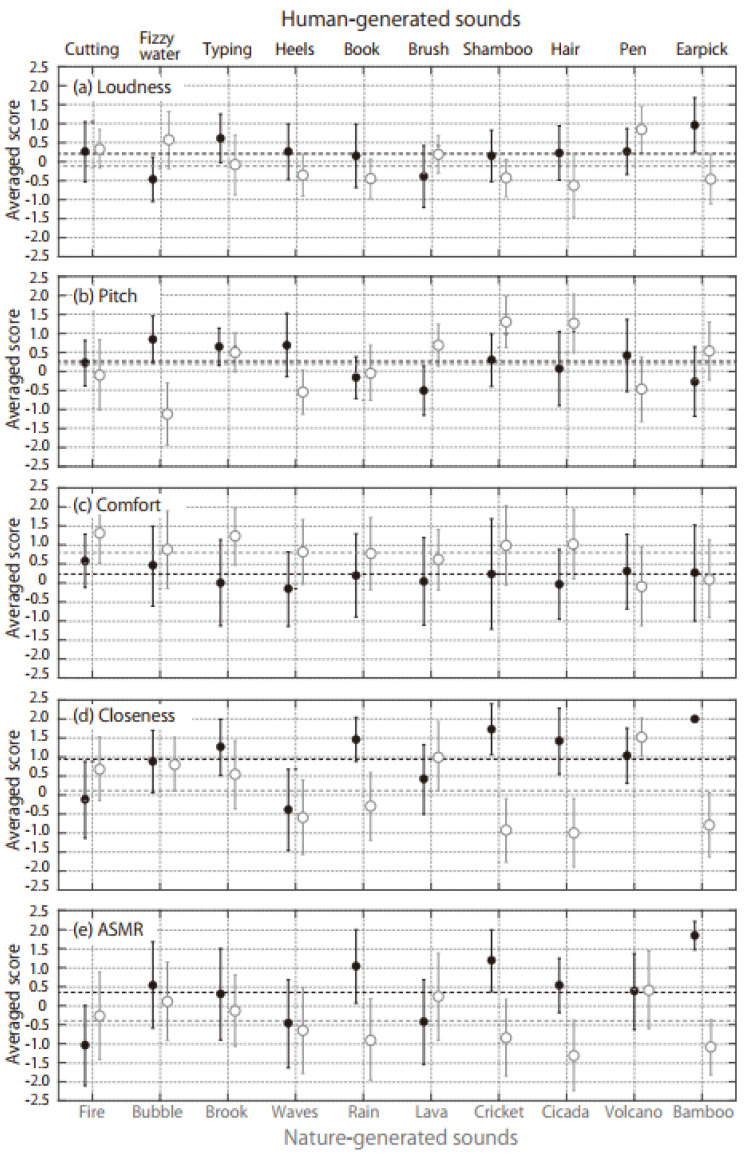
Average scores for (**a**) loudness, (**b**) pitch, (**c**) comfort, (**d**) closeness, and (**e**) the ASMR. Black and gray symbols are results for human- and nature-generated sounds, respectively. The bar on each symbol shows standard deviations. The black and gray horizontal dot lines are total averaged scores for human- and nature-generated sounds, respectively.

Figure 2 shows the average scores of the subjective loudness, pitch, comfort, closeness, and ASMR for the human- (black symbols) and nature-generated (gray symbols) sounds. The subjective loudness, closeness, and ASMR scores tended to be higher for the human-generated sounds than for the nature-generated sounds. According to a *t*-test of the total scores of the human- (260 = 10 ASMR triggers × 26 participants) and nature-generated (260) sounds, there were significant differences in the subjective loudness (*t*_338_ = 3.65, *p* < 0.01), closeness (*t*_338_ = 8.69, *p* < 0.01), and ASMR (*t*_338_ = 7.84, *p* < 0.01). In contrast, the comfort was higher for the nature-generated sounds (*t*_338_ = 6.28, *p* < 0.01) and there was no significant difference in the perceived pitch between the nature- and human-generated sounds (*t*_338_ = 0.28, *p* = 0.78). The three sounds with the highest ASMR values were Earpick, Shampoo, and Book for the human-generated sounds and Volcano, Lava, and Bubble for the nature-generated sounds, and they were commonly perceived to be close. The three sounds with the lowest ASMR values were Cutting, Heels, and Brush for the human-generated sound and Cicada, Bamboo, and Rain for the nature-generated sounds, and they were commonly perceived to be far.

**Figure 3:**
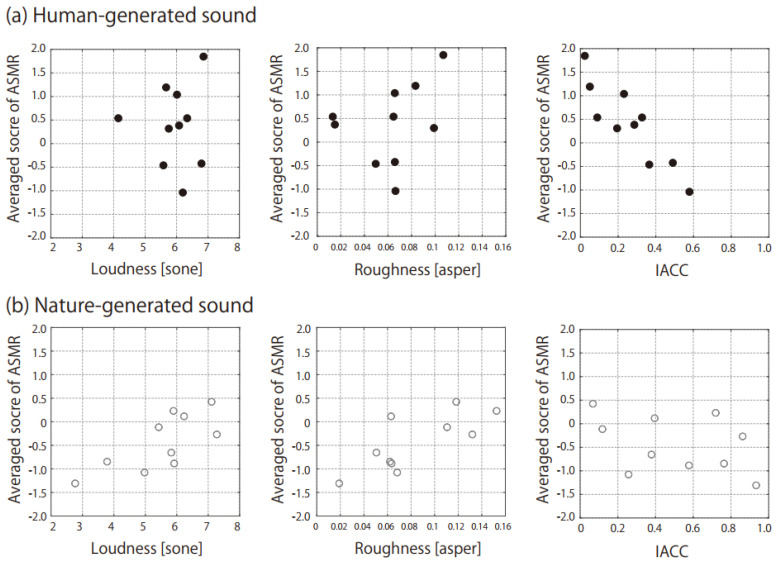
Relationships of the ASMR scores with loudness, roughness, and IACC for (**a**) human-generated sounds (black symbols) and (**b**) nature-generated sounds (gray symbols).

Table 2 shows the Pearson correlation coefficients of the ASMR scores with the sound quality parameters that had normal distributions. The ASMR scores of the nature-generated sounds were strongly correlated with loudness and roughness among the Zwicker parameters. Meanwhile, the ASMR scores of the human-generated sounds were strongly correlated with the IACC among the ACF/IACF parameters. Figure 3 shows the ASMR scores as functions of loudness, roughness, and IACC which showed high Pearson correlation coefficients. The strong negative relationship could be observed in the IACC for the human-generated sounds, while the positive relationships could be observed in the loudness and roughness for the nature-generated sounds. Table 2 lists the correlation coefficients of the ASMR scores with the scores of the other psychological judgements, too. The subjective loudness had a high correlation with the ASMR generated by the nature-generated sounds. Additionally, closeness had a high correlation with the ASMR generated by both human- and nature-generated sounds.

## 4. Discussion

The primary reason why the ASMR scores of the human-generated sounds were significantly higher than the nature-generated sounds may be the distance from the sound source to the receiver. In fact, the perceived closeness was strongly related to the ASMR sensation (Table 2). The human-generated sounds were recorded at a position close to the binaural devices whereas the nature-generated sounds were recorded at a certain distance from the sound source. Additionally, the ASMR triggers used in previous studies (e.g., whisper voice, personal attention, and crisp sounds) were recorded close to the binaural microphone [1,9,10,11,12,13]. In these triggers, the personal attention refers to role-play videos that concentrate on the viewer, so that it is not just an ASMR trigger but the scenario/context in which the triggers occur. To examine acoustical aspects in the triggers, sounds including the scenario/context (e.g., speech) were removed from the triggers used in this study. However, the Earpick, Shampoo, and Hair sounds that had high ASMR scores made the participants imagine to be acted upon themselves. It seems undeniable that such unintended personal attention might help the ASMR sensations for these triggers, and the very closed triggers to the participants are likely to induce the pseudo-personal attention.

For nature-generated sounds, sound qualities relating to higher loudness and roughness induced the ASMR experience (Figure 3). These parameters also had high correlations with the closeness scores (loudness: *r* = 0.73, *p* < 0.05, roughness: *r* = 0.77, *p* < 0.01). The nearby sounds produce the ASMR, whereas some listeners are annoyed by sounds close to their ears. Therefore, the comfort scores were significantly lower for the human-generated sounds (Figure 2c). Although it is well known that people who experience ASMRs report feeling relaxed and sleepy after watching and listening to ASMR content, some people feel annoyance from the triggers [4]. The hypersensitivity for the auditory perception is the same origin for the ASMR and misophonia; however, higher-order cognitive processing may divide expressed emotions into the preference for the ASMR or annoyance for the misophonia [3]. The very closed sound makes the listeners imagine either the positive personal attention or negative invasion of territory. Separation at the cognitive processing may be related to the different interpretation of the closeness. If this study contains speech signals addressing the participants, the comfort scores for the human-generated sounds may be improved.

Although a previous ASMR study reported that sounds with a lower pitch were more likely to produce an intense ASMR sensation [9], the pitch scores and ACF/IACF parameters relating to pitch (i.e., τ_1_, ϕ_1_ and W_Φ(0)_) did not affect the ASMR score (Figure 2b and Table 2). The bass or low-frequency response is higher when a sound source is close to a directional or cardioid microphone (in what is known as the acoustical proximity effect) [31]. In this study, the acoustical proximity effect might occur to the same degree for any human-generated sound that is sufficiently close to the binaural microphones.

The human-generated sounds with a lower IACC produced a stronger ASMR sensation (Figure 3). The IACC is related to the spatial characteristics of a sound field, and it can thus control the location of a sound image. In concert halls (having a diffused sound field), the IACC is lower when the distance between the sound source and receiver is greater [32], because the direct sound that tends to increase the IACC is weakened relative to reflections and reverberations. In contrast, in laboratory experiments, the IACC can be controlled by changing the interchannel phase difference of stereo loudspeakers in front of the listener, and a sound with lower IACC can generate a sound image closer to the listener (in what is referred to as auditory distance rendering) [33,34,35,36,37]. This phenomenon observed in auditory distance rendering agrees with the results of the present study. However, the binaural phase of the ASMR triggers used in this study was not manipulated digitally; therefore, there may be another explanation in this case. The IACC indicates the similarity of time-varied sounds entering the left and right ears. It is thus expected that sound near one ear (e.g., the sound heard when using an earpick) has low similarity (low IACC) between the ears, and we thus have to separate the relationships between the IACC and the distance from the sound image into near and far fields centering around the listener’s head.

Finally, we discuss the possible applications of these findings in clinical treatments for misophonia. The most successfully used treatment at the clinical scene is cognitive behavioral therapy (CBT) [38,39,40,41,42]. The CBT protocol constitutes four different techniques: task concentration exercises, counterconditioning, stimulus manipulation, and relaxation exercises. Following treatment, 48% of the patients showed a significant reduction of misophonia symptoms [43]. In a session of stimulus manipulation, the patients are instructed to change the pitch and time interval of sound triggers by using an audio-editing software, and this manipulation initiates a sense of control over their personal misophonic trigger sounds. In this study, the IACC is the most effective factor to control the ASMR sensation, so the change of IACC (e.g., convolution with binaural impulse responses) may be effective to let the patients know the misophonic trigger sounds under their control.

## 5. Conclusions

The following conclusions are drawn from the results of the study.

(1) Human-generated sounds are more likely to trigger stronger ASMRs than nature-generated sounds.
(2) Among possible ASMR auditory triggers, sounds perceived to be close to the listener are more likely to evoke the ASMR sensation.
(3) In the case of nature-generated sounds, the ASMR triggers with higher loudness and roughness among Zwicker parameters are more likely to evoke the ASMR sensation.
(4) In the case of human-generated sounds, the ASMR triggers with a lower IACC among the ACF/IACF parameters are more likely to evoke the ASMR sensation.

## Figures and Tables

**Table 1:** Human- and nature-generated sounds and calculated Zwicker’s and ACF/IACF parameters.

Sound Source			Zwicker’s Parameters				ACF/IACF Parameters			
	Short Title	Contents	Loudness [sone]	Sharpness [acum]	Roughness [asper]	Fluctuation Strength [vacil]	τ_1_ [ms]	ϕ_1_	W_Φ(0)_ [ms]	IACC
Human-generated sound	Cutting	Cutting vegetable	6.20	1.63	0.07	1.31	2.52	0.20	0.26	0.58
	Fizzwater	Stirring carbonated water	4.15	3.25	0.06	0.02	0.22	0.29	0.06	0.09
	Typing	Typing a keyboard	5.75	2.22	0.10	0.59	0.86	0.15	0.09	0.19
	Heels	Footsteps of high heels	5.58	1.58	0.05	0.43	1.56	0.19	0.36	0.37
	Book	Flipping a book	6.01	1.94	0.07	0.06	1.40	0.13	0.13	0.23
	Brush	Brushing something	6.79	1.78	0.07	0.05	1.99	0.15	0.14	0.49
	Shampoo	Washing hair with shampoo	5.67	2.33	0.08	0.33	1.92	0.04	0.10	0.05
	Hair	Cutting hair	6.34	2.17	0.01	0.39	0.93	0.42	0.09	0.33
	Pen	Writing with pen	6.08	2.54	0.01	0.39	0.42	0.29	0.06	0.29
	Earpick	Earpick	6.86	1.30	0.11	0.74	6.45	0.05	0.40	0.02
Nature-generated sound	Fire	Building a fire	7.28	1.88	0.13	0.03	3.32	0.11	0.12	0.86
	Bubble	Bubbles under water	6.23	0.70	0.06	0.07	6.74	0.21	0.77	0.40
	Brook	Murmur of a brook	5.43	1.87	0.11	0.07	1.70	0.13	0.15	0.12
	Waves	Sound of waves	5.83	1.43	0.05	0.06	3.63	0.05	0.30	0.38
	Rain	Sound of rain	5.92	2.11	0.06	0.10	3.63	0.05	0.30	0.58
	Lava	Lava flowing	5.90	2.53	0.15	0.02	0.68	0.09	0.07	0.72
	Cricket	Bell-ringing cricket	3.78	3.19	0.06	0.02	0.48	0.84	0.07	0.76
	Cicada	Evening cicada	2.77	2.69	0.02	0.02	0.28	0.95	0.09	0.93
	Volcano	Bubbles of mud volcano	7.11	1.46	0.12	0.29	1.65	0.15	0.22	0.07
	Bamboo	Wind through bamboo forest	4.98	3.13	0.07	0.06	3.76	0.02	0.06	0.26

**Table 2:** Correlation coefficients of the ASMR scores among Zwicker’s parameters, ACF/IACF parameters and subjective judgements (**: *p* < 0.01, *: *p* < 0.05).

	Zwicker’s Parameters				ACF/IACF Parameters				Subjective Judgements			
	Loudness	Sharpness	Roughness	Fluctuation Strength	τ_1_	ϕ_1_	W_Φ(0)_	IACC	Subjective Loudness	Pitch	Comfort	Closeness
ASMR (Total)	0.42	−0.21	0.27	0.15	0.12	−0.36	0.06	−0.67 **	0.64 **	−0.29	−0.38	0.93 **
ASMR (Human)	0.04	0.11	0.32	−0.30	0.39	−0.32	−0.04	−0.89 **	0.38	−0.20	0.02	0.93 **
ASMR (Nature)	0.73 *	−0.61	0.77 **	0.47	0.14	−0.46	0.34	−0.41	0.92 **	−0.53	−0.17	0.96 **

## Data Availability

Not applicable.
